# Identifying Key Biomarkers and Immune Infiltration in Female Patients with Ischemic Stroke Based on Weighted Gene Co-Expression Network Analysis

**DOI:** 10.1155/2022/5379876

**Published:** 2022-04-08

**Authors:** Haipeng Xu, Kelin He, Rong Hu, YanZhi Ge, Xinyun Li, Fengjia Ni, Bei Que, Yi Chen, Ruijie Ma

**Affiliations:** ^1^The Third School of Clinical Medicine (School of Rehabilitation Medicine), Zhejiang Chinese Medical University, Key Laboratory of Acupuncture and Neurology of Zhejiang Province, Hangzhou, Zhejiang, China; ^2^Department of Acupuncture and Moxibustion, Third Affiliated Hospital of Zhejiang Chinese Medical University, Hangzhou, Zhejiang, China; ^3^The First Clinical Medical College, Zhejiang Chinese Medical University, Hangzhou, Zhejiang, China

## Abstract

Stroke is one of the leading causes of death and disability worldwide. Evidence shows that ischemic stroke (IS) accounts for nearly 80 percent of all strokes and that the etiology, risk factors, and prognosis of this disease differ by gender. Female patients may bear a greater burden than male patients. The immune system may play an important role in the pathophysiology of females with IS. Therefore, it is critical to investigate the key biomarkers and immune infiltration of female IS patients to develop effective treatment methods. Herein, we used weighted gene co-expression network analysis (WGCNA) to determine the key modules and core genes in female IS patients using the GSE22255, GSE37587, and GSE16561 datasets from the GEO database. Subsequently, we performed functional enrichment analysis and built a protein-protein interaction (PPI) network. Ten genes were selected as the true central genes for further investigation. After that, we explored the specific molecular and biological functions of these hub genes to gain a better understanding of the underlying pathogenesis of female IS patients. Moreover, the “Cell type Identification by Estimating Relative Subsets of RNA Transcripts (CIBERSORT)” was used to examine the distribution pattern of immune subtypes in female patients with IS and normal controls, revealing a new potential target for clinical treatment of the disease.

## 1. Introduction

Stroke is one of the leading causes of death and long-term disability in the world. Every year, it is estimated that 96 million new cases of ischemic stroke and 41 million new cases of hemorrhagic stroke are diagnosed worldwide [1]. Arterial occlusion-related ischemic stroke is a major cause of the majority of strokes, accounting for 87 percent of stroke cases and nearly half of all deaths [2]. Current evidence shows that [3] the focus of IS treatment is on timely blood clot removal and long-term secondary prevention. However, the molecular mechanism of IS remains unknown. In addition, gender differences in stroke patients may influence clinical manifestations, epidemiological features, pathophysiology, prognoses, and outcomes. Therefore, a study of stroke patients with no gender differences may result in biased results [4, 5]. Previous research indicates that [6] women had a higher risk of stroke-related death than men, with six out of ten women dying from stroke. This increased risk could be attributed to a variety of factors, one of which is that women live averagely longer, which increases their risk of stroke. Meanwhile, other risk factors such as high blood pressure during pregnancy and certain types of birth control medication increase their overall risk of stroke [7]. As such, we only used data of female IS patients.

Researchers frequently use WGCNA to investigate the complex relationships between genes and phenotype; it converts gene expression data to co-express module, allowing a better understanding of the network signal responsible for phenotypic characteristics [8]. This approach has been widely used in a variety of biological processes whereby it plays a significant role in the comparison of differentially expressed genes and the discovery of co-expression module genes [9]. The WGCNA approach has provided functional interpretation tools in system biology, and it is widely used in stroke-related research [10–12]. In this study, we used genes in three GEO datasets from female patients with ischemic stroke and healthy controls to build a co-expression network by WGCNA. In addition, we looked at the relative proportions of 22 different types of immune cells in 72 blood samples from IS patients and 24 blood samples from healthy females. We built co-expression modules based on the expression data of female IS patients and perform integrated bioinformatics analysis of the modules of interest, whose gene expression was specifically co-related to female IS patients and could provide potential therapeutic targets for disease management.

## 2. Materials and Methods

### 2.1. Dataset Information

The gene expression data used in this study were obtained from the Gene Expression Omnibus (GEO) database (https://www.ncbi.nlm.nih.gov/geo/) [13]. Data processing was divided into three stages. First, the one probe expression matrix files downloaded from the GEO database were normalized and log2 transformed. The platform annotation file was then matched with each probe expression matrix, and only well-annotated probes were retained. In order to ensure the accuracy of included data, we analyzed the average expression values of multiple probes corresponding to a gene. Finally, R package sva, installed from Bioconductor (https://bioconductor.org/), was applied to eliminate the heterogeneity caused by different experimental batches and platforms.

### 2.2. Construction of Co-Expression Network

The WGCNA method assists in investigating gene set expression. Herein, the WGCNA R package was used at various stages for the construction and module division of different gene networks through the following major steps [14]. The samples were clustered to see if any obvious outliers were present. Next, automatic network construction was used to construct the co-expression network. Hierarchical clustering and a dynamic tree-cutting function detection module were employed. Gene salience (GS) and module membership (MM) were calculated to correlate modules with clinical characteristics. The module with the largest absolute value of Pearson's correlation of module membership (MM) and a *p*-value <0.05 was defined as the hub module. MM >0.8 and GS >0.2 represented high module connectivity and high clinical significance, respectively. The corresponding module gene information was extracted for further analysis.

### 2.3. Functional Enrichment Analysis

The intersection genes of target modules were extracted from the network, and the enrichment analysis was performed to further investigate the functions of each module. Gene Ontology (GO) terms and the Kyoto Encyclopedia of Genes and Genomes (KEGG) pathways were considered to be enriched with thresholds of p-value <0.05 and an enriched gene count >2.

### 2.4. Immune Infiltration through CIBERSORT Analysis

CIBERSORT is a deconvolution algorithm that can analyze any immune cell subtype and accurately quantify the various immune cell components. We looked at analyzed immune infiltration in 72 female patients with ischemic stroke and 24 healthy females. These immune cells include immature B cells, memory B cells, and the other 19 types of immune cells. The percentage of each immune cell in the sample was calculated, with *p* < 0.05. The R software packages “ggplot 2” and “GGPUBR” were used to compare the levels of immune cell infiltration in female stroke patients and normal female subjects.

### 2.5. Protein-Protein Interaction (PPI) Network Analysis

Genes were imported into the STRING website (version: 11.0) to explore the mutual relationship between proteins encoded by different genes [15]. We ensured that the lowest interaction score is greater than 0.4, and isolated nodes in the network were removed. The analysis results were output to a TSV format file, and Cytoscape software (version: 3.7.1) was used for details processing and module analysis. Cytohubba is a plug-in that can find closely connected nodes in a complex network based on topology. It can be downloaded from the Cytoscape App Store. We used this plug-in to find the most significant cluster of 10 nodes in a PPI network using the default parameters.

### 2.6. Animal Experiments

Female SD rats (3-4 months old); mean body weight =220 g) were provided by Shanghai Xipu Bikai experimental animal company (animal license No: SCXK (Shanghai) 2018-0006). Animal experiments were performed following the China legislation on the use and care of laboratory animals and were approved by the Medical Norms and Ethics Committee of Zhejiang Chinese Medical University. Female SD rats were randomly assigned to the normal group and the IS group (*n* =8 per group).

### 2.7. Model Preparation

The middle cerebral artery occlusion (MCAO) model was established in 4-month-old female SD rats. First, the rats were anesthetized with 3% pentobarbital and then fixed on an operating table. A midline neck incision was used to expose the common carotid artery (CCA), external carotid artery (ECA), and internal carotid artery (ICA). A 6-0 nylon suture was inserted into the internal carotid artery through the external carotid artery stump and gently advanced to occlude the middle cerebral artery. Sham-operated animals underwent the same surgical operation but with no nylon suture insertion. Following that, the wound was then sutured and disinfected. All animals received intraperitoneal injection of penicillin (100 U/d) to prevent infection.

### 2.8. Quantitative Reverse Transcription PCR (qRT-PCR)

qRT-PCR was used to confirm the expression of 10 hub genes in peripheral blood. qPCR was performed on the CFX96 Real-Time System (BioRad, USA) using the Fast Start Universal SYBR Green Master kit (TaKaRa Bio Inc., China) according to the manufacturer's protocol. The relative quantification was performed by the *ΔΔ*CT method. Primer sequences are listed in Table 1.

### 2.9. Statistical Analyses

All statistical analyses between control and experimental groups were completed using the GraphPad Prism8 and data were analyzed with a one-way ANOVA followed by Tukey Kramer tests. The results are presented as mean ± SEM; *p* < 0.05 denote statistical significance.

## 3. Results

### 3.1. Workflow

**Figure 1:**
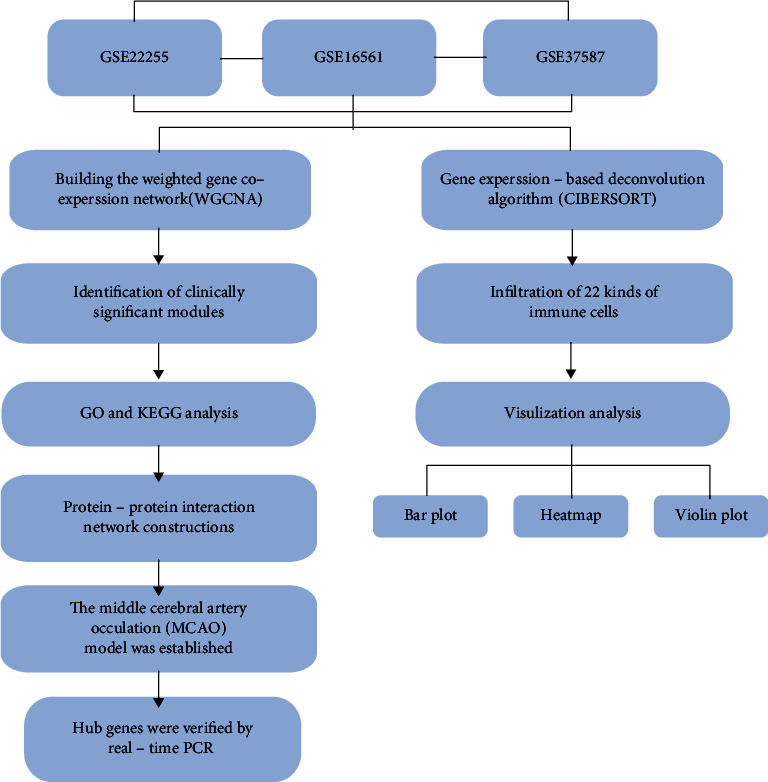
The workflow of the whole study.

Figure 1 shows a schematic diagram of the workflow. A co-expression network was built in a sample of female IS patients and a sample of normal females, and several modules of clinical significance were identified. The function of the key modules was then examined to reveal the core differential genes in female stroke patients.

### 3.2. Constructing the Weighted Gene Co-Expression Network

**Figure 2:**
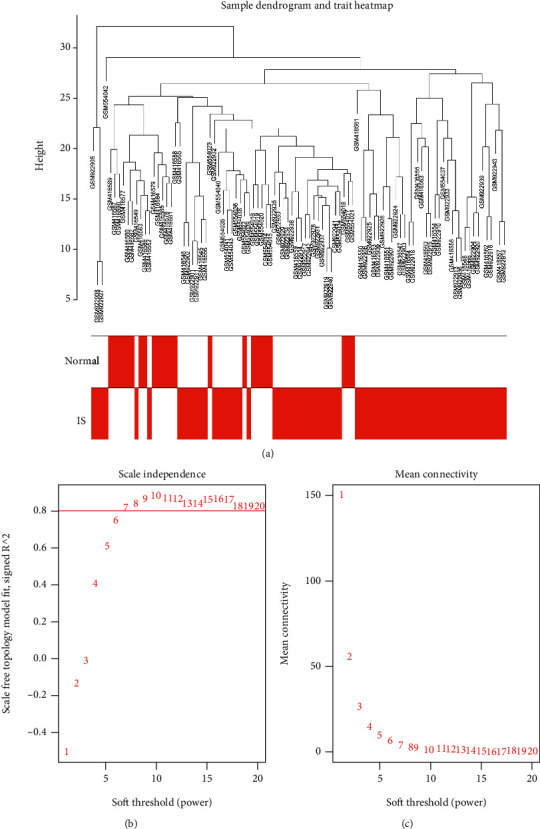
Construction and module division of the co-expression network. (a) Cluster tree diagram of the sample. The cluster tree reflects the distance between 96 samples. (b) Soft threshold (*R*^2) determination; the fitting degree *R*^2 increases with an increase in the soft threshold. When the fitting degree *R*^2 > 0.8 (red line), the corresponding network is more consistent with the scale-free network distribution. (c) Soft threshold selection (average connectivity).

The GSE22255, GSE37587, and GSE16561 datasets were obtained from the GEO database. A total of 24 normal samples and 72 IS samples were examined. The samples were first clustered, and the obvious outlier samples were eliminated by setting the threshold as illustrated in Figure 2(a). The sample cluster tree included 96 samples. After that, we selected the soft threshold. As illustrated in Figures 2(b) and 2(c), when *R*^2 > 0.8, the fitting degree was high enough and the mean connectivity was relatively high. Furthermore, we used the WGCNA R software package to construct the gene network and determined the module based on a certain soft threshold. A weighted gene co-expression network was built to split the cluster, and the co-expression modules were divided using dynamic cutting and module merging.

### 3.3. Identification of Clinically Significant Modules

**Figure 3:**
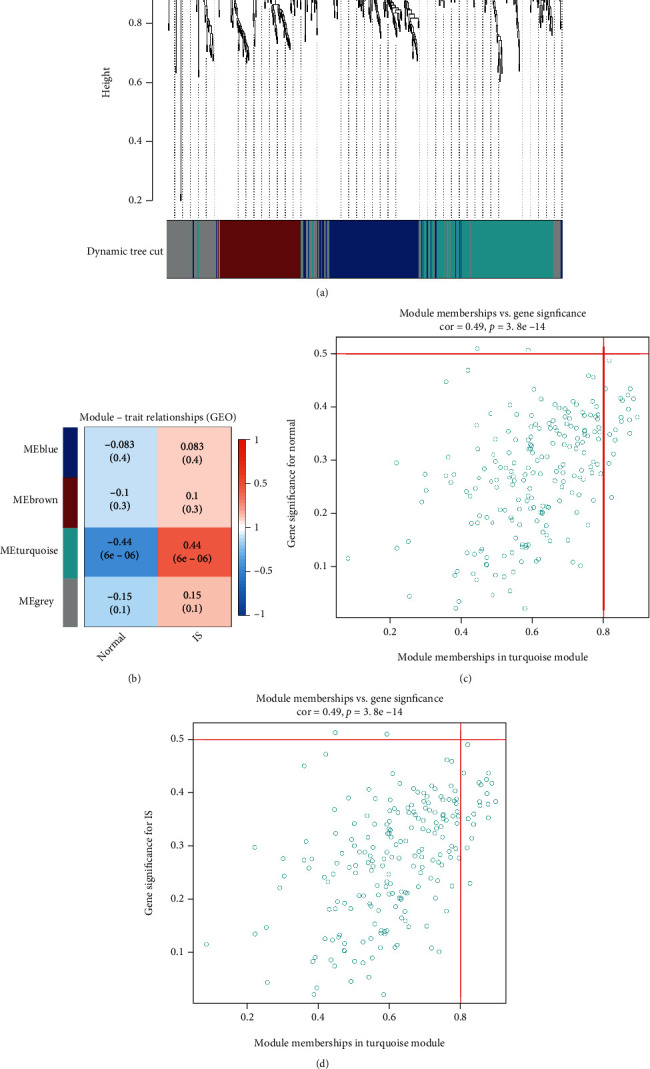
Identification of modules associated with clinical characteristics of IS females. (a) Cluster tree diagram and module feature relationship diagram for hierarchical cluster analysis to detect co-expression clustering with the corresponding color distribution. Each color represented a module in the gene co-expression network constructed by WGCNA. Heat map of the correlation between the module. (b) Characteristic gene in IS female samples and normal samples. ((c) and (d)) The relationship between the scatter plot of the members of the module and the genetic importance of morbid states.

The correlation analysis of gene expression and disease characteristics was performed by WGCNA, and four-gene expression modules were developed (Figure 3(a)). Next, we linked modules to clinical features and searched for the most important ones. As demonstrated in Figures 3(b) and 3(c), gene expression of the turquoise module was most closely related to the two groups of key factors (normal and IS) (*r* =0.44). Therefore, we selected the turquoise module for the subsequent analysis.

### 3.4. Functional Analysis of the Key Module

**Figure 4:**
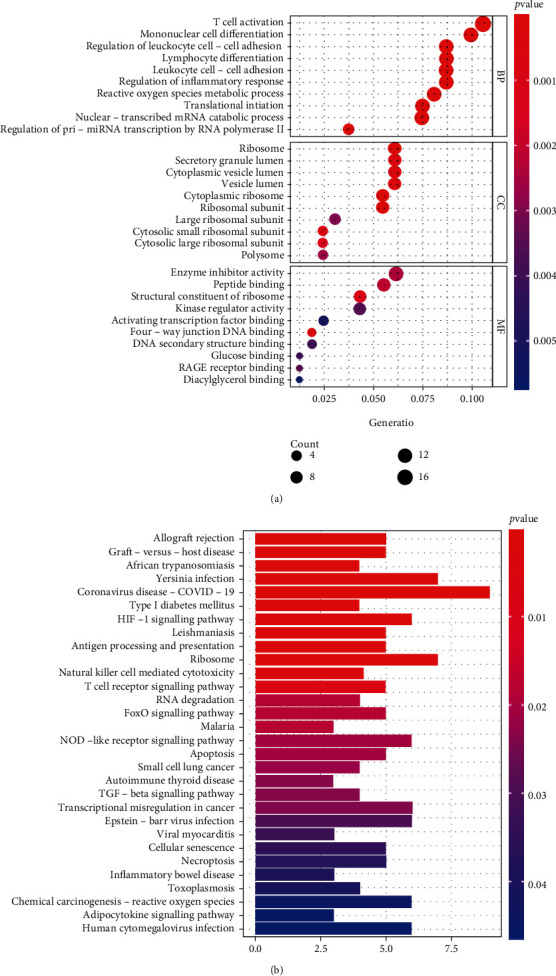
GO and KEGG pathway analysis. (a) GO analysis of genes involved in turquoise module. (b) Enriched pathways of genes in turquoise module by the KEGG.

GO and KEGG analyses were performed on the turquoise module genes. The GO analyses results demonstrated that the genes were primarily related to the regulation of T cell activation, mononuclear cell differentiation, and neutrophil-mediated immunity in biological processes (BP) (Figure 4(a) and Table 2). Furthermore, gene cell components were primarily enriched in the ribosome and secretory granule lumen. In terms of molecular function (MF), genes were primarily enriched in enzyme inhibitor activity and ribosome structural constituents. Next, we performed functional analysis (KEGG analysis) on the genes in the turquoise module (Figure 4(b) and Table 3). The findings revealed an association between the regulation pathway of the turquoise module with the ribosome, the HIF−1 signaling pathway, and natural killer cell-mediated cytotoxicity.

### 3.5. Immune Infiltration Analyses

**Figure 5:**
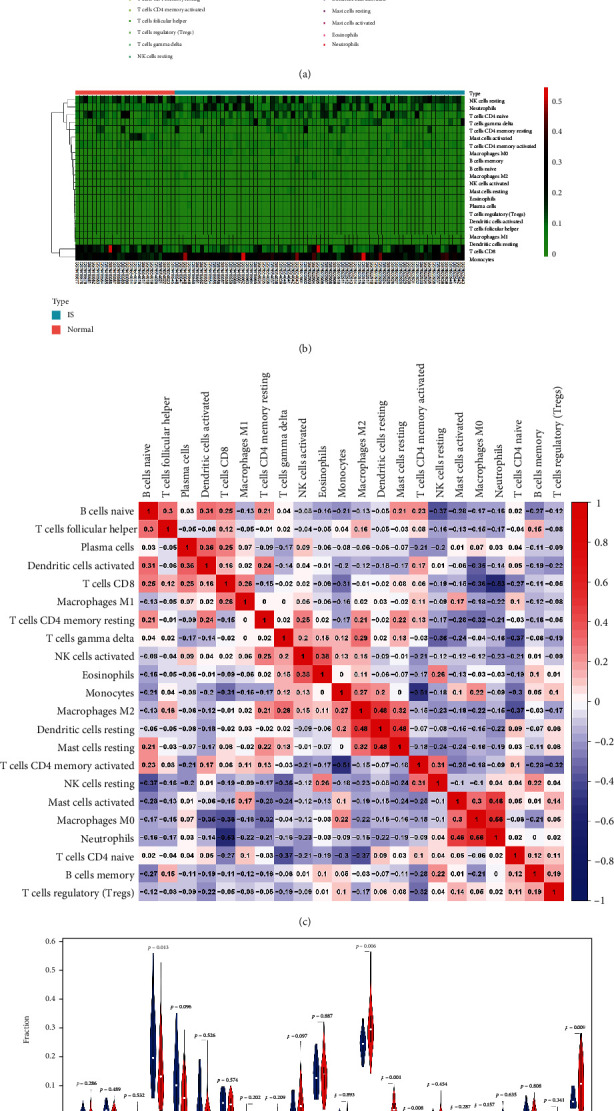
Immunoinfiltration of IS female and healthy female. (a) Percentage distribution of 22 immune cell subtypes in 96 samples from three datasets. (b) Heat map of the ratio of 22 immune cells in each sample. (c) Related heat maps of 22 immune cells. (d) Violin diagram of the difference in immune cell infiltration between female stroke patients and normal females.

**Figure 6:**
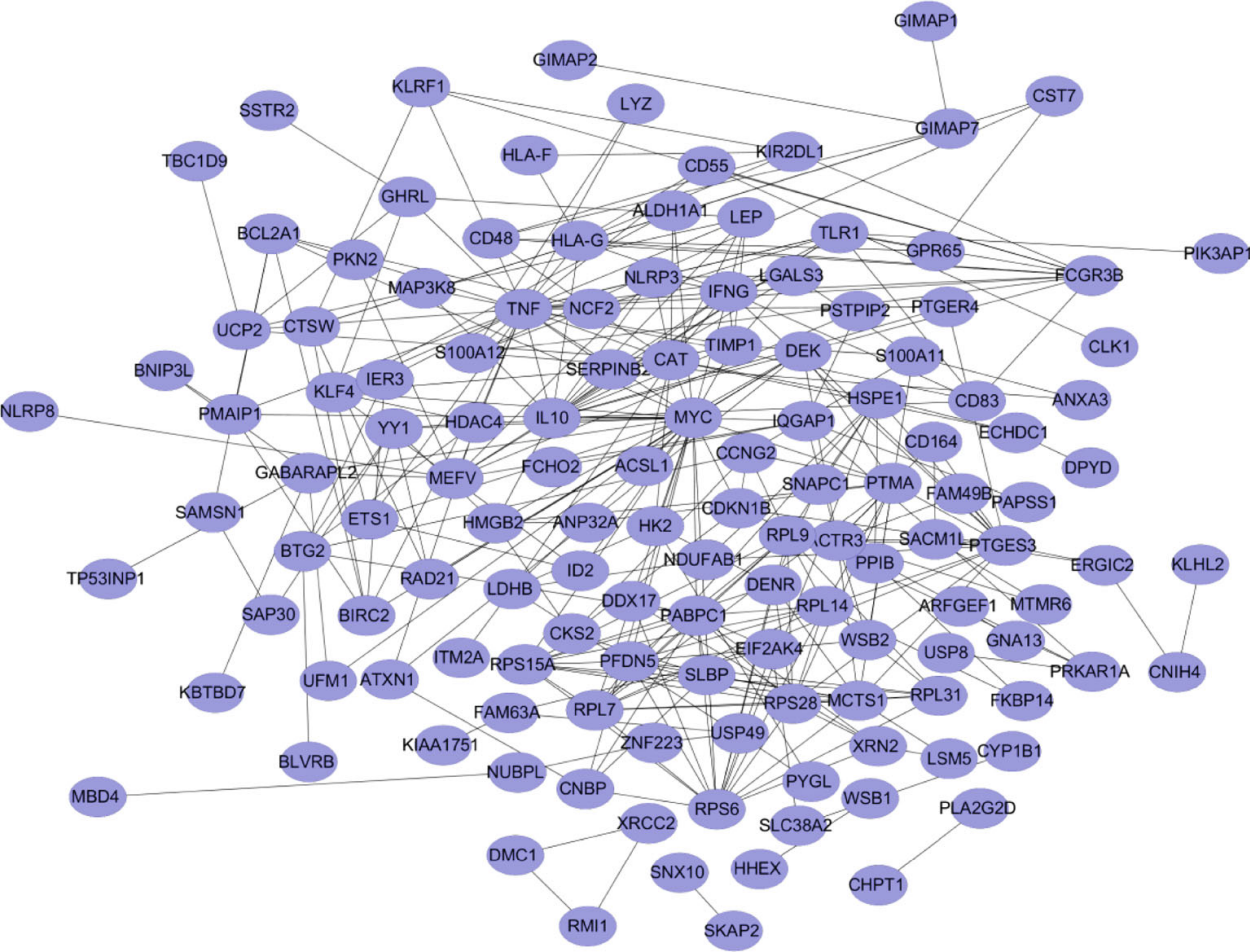
Protein-protein interaction (PPI) networks associated with differences in female patients with IS.

We explored the difference in immune infiltration of 22 immune cell subsets between female IS patients and healthy females. The histogram illustrated in Figures 5(a) and 5(b) depicts the distribution of various immune cells in the sample. The various colors represent different types of immune cells. The main immune infiltration cells included neutrophils, NK cells resting, macrophages M0, T cells CD8, and monocytes. The proportions of various infiltrated immune cell subsets were weakly moderately correlated (Figure 5(c)). F Neutrophils and macrophages M0, for example, was 0.48, and T cells CD8 and neutrophils was -0.53, and so on. Moreover, IS females had more monocytes and neutrophils when compared to healthy females (Figure 6(d)).

### 3.6. Identification of Hub Genes in the Functional Modules and Protein-Protein Interaction Network Construction

**Figure 7:**
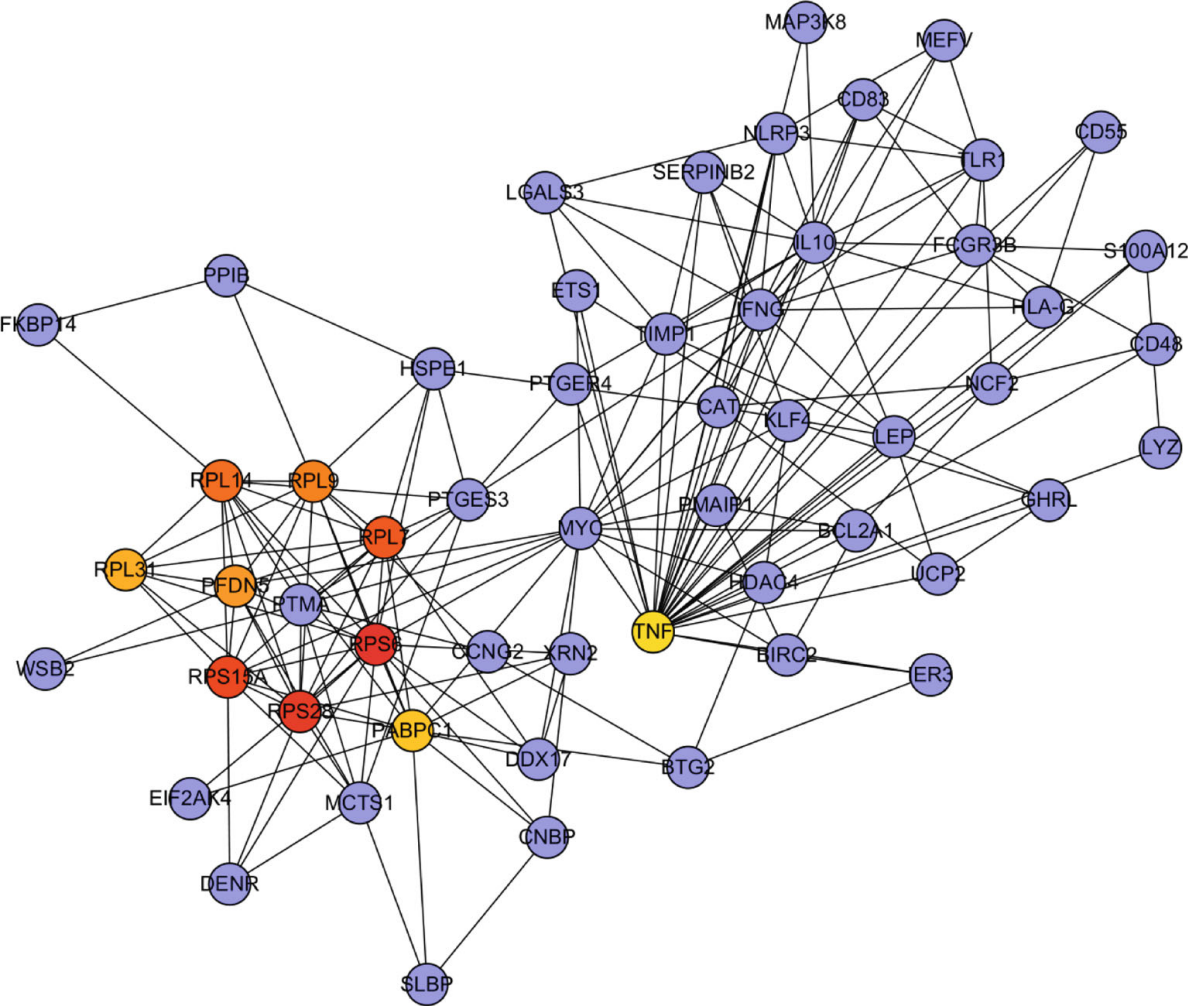
Core gene clusters in the co-expression network constructed from female IS patients. The depth of color indicated the levels of the central genes from low to high.

A total of 211 genes were identified from the clinically significant module (turquoise module) of the co-expression network. A PPI network was then constructed using the STRING database (Figure 6). Finally, cytoHubba was used to screen for and visualize the hub genes (RPS15A, RPS6, RPS28, RPL7, PABPC1, RPL31, RPL14, RPL9, PFDN5, TNF) in the network (Figure 7).

### 3.7. Validation of the Hub Genes Using qRT-PCR

**Figure 8:**
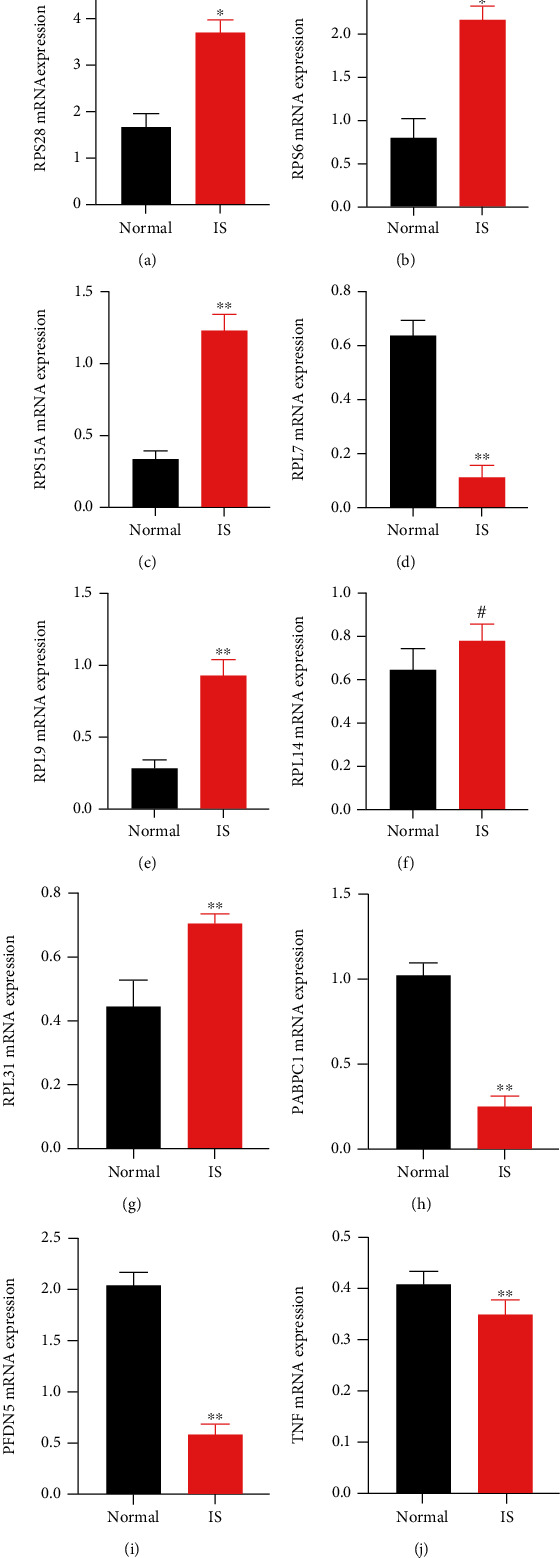
Validation of the hub genes by qRT-PCR. Black, normal samples; red, IS samples. ∗∗means *p*-value <0.01, and #means no difference. All data correspond to the average ± SEM. Statistical significance was assessed by the two-tailed Student's *t*-test.

qRT-PCR was used to reverify the 10 hub genes to further demonstrate the reliability of the WGCNA results. First, the stroke model was constructed by performing MCAO as described in the methods. Next, the peripheral blood was used for qRT-PCR. The results showed that the expression of the RPS28, RPS6, RPS15A, RPL9, TNF, and RPL31 genes were significantly higher in the IS model, whereas the expression of RPL7, PABPC1, and PFDN5 genes was significantly lower in the IS model (*p*-value<0.01). However, no statistically significant differences in expression levels were found between the two groups (Figure 8(f)).

## 4. Discussion

Ischemic stroke, a neurological disease with a high morbidity and mortality rate, is one of the leading causes of permanent disability in adults [16]. Previous evidence indicates that [17] the etiology, risk factors, and prognosis of this disease all differ by gender. Of note, the risk of IS increases with an increase in the life expectancy of women. Ischemic stroke is also a common female complication during pregnancy and puerperium. Nearly 30 cases per 100000 cases of gestation (including all subtypes) show stroke symptoms in the two periods, with puerperae in high-risk groups having a higher incidence of IS [18]. In this view, females may bear a heavier burden than males.

Three dates were used in this study to collect 96 peripheral whole blood samples, 72 IS females and 24 normal females. We built a co-expression module using three datasets from WGCNA and confirmed that the turquoise module was crucial for females with IS (Figures 2 and 3). GO analysis demonstrated that immune response and ribosomes played critical roles in the pathogenesis of females with IS. Furthermore, KEGG analysis revealed that the main pathways of this disease in IS female patients were ribosome, HIF−1 signaling pathway, and natural killer cell-mediated cytotoxicity (Figure 4 and Tables 2 and 3). Ribosomal proteins (RPs) play an important role in the regulation of gene expression and protein synthesis [19]. Elsewhere, an experimental study found that cytoplasmic ribosomes play a role in functional recovery after ischemic stroke [20]. In the present study, the GO and KEGG results revealed that immune response and ribosomes are critical links in the mechanism of female patients with ischemic stroke.

Based on the above findings, we looked into the difference in immune infiltration of 22 immune cell subsets between IS females and healthy females. Mechanistically, the immune system is activated following ischemic stroke. A series of changes occur during immune cell migration to the ischemic area, resulting in either beneficial or detrimental effects on ischemic outcomes [21]. More importantly, certain key immune cells could become a new target for IS prevention or treatment. Our findings demonstrated that neutrophils, NK cells resting, macrophages, and T cells CD8 were the primary immune infiltrating cells.

Previous research has shown [22] that neutrophil infiltration is involved in recruitment of other immune cells. While some studies suggest that neutrophil infiltration may exacerbate ischemic stroke injury, other studies have discovered [23, 24] neutrophil involvement in tissue remodeling after stroke. NK cells are large granular lymphocytes of innate immunity that are required for IS immunosurveillance. NK cells regulate cellular immune response by blocking CD8 + T cell activation [25]. Infiltration of NK cells into the periinfarct area, on the other hand, can hasten neuronal death [26]. Improving NK cell infiltration into the periinfarct area is thus an important therapy for female patients with ischemic stroke that will eventually improve clinical responses. Macrophages are critical regulators of host defense in organisms, and they play critical roles in the repair of the central nervous system. In addition to providing neuroprotection, macrophages are a major source of proinflammatory cytokines [27]. These proinflammatory factors prevent the repair of brain tissue [28]. T cells regulate both innate and adaptive immunity, which may play a role in the pathogenesis of some neurological diseases [29]. Previous evidence shows that [30] activated and infiltrated microglia/macrophages after cerebral ischemia can stimulate activated CD4 + T cells to differentiate into Th1 or Th2 cells, which then produce proinflammatory or anti-inflammatory cytokines to damage or protect the brain. On the other hand, CD8+ cytotoxic T cells cause neuronal death and exacerbated brain injury via cell interaction. Findings from the present study demonstrate that T cells, macrophages, NK cells, and neutrophils may be important immune targets for the treatment of female ischemic stroke patients. The PPI network yielded 10 hub genes of female IS patients, which is consistent with the results of the GO and KEGG analyses.

Ribosomes have a wide range of complex functions. They are made up of several ribosomal proteins (RP), ribosomal RNA (rRNA), and small nucleolar RNA [31]. In terms of functions, ribosomal proteins are involved in a variety of critical biological processes in diseases. RPS15A (ribosomal protein S15A) is a component of the ribosomal 40s subunit that is involved in a series of biological processes, including proliferation, apoptosis, differentiation, and DNA repair [32]. Increasing evidence suggests that [33] RPS15A exerts critical functions in the development and progression of cancer. Ribosomal protein S6 (RPS6), a component of the cell translation system, has been shown to play a role in the progression of 40s ribosome biogenesis [34]. rpS6 phosphorylation is commonly used as a marker of neuronal activity in neuroscience [35]. Recent evidence shows that [36] RPS28 regulates MHC Class I peptide generation for immunosurveillance. However, the dysfunction of RPS15A, RPS6, and RPS28 in females with ischemic stroke patients has never been reported. Similarly, the present study found upregulated levels of RPS15A, RPS6, and RPS28 in IS samples (Figures 8(a)–8(c)).

Ribosomal protein S7 (RPL7), ribosomal protein S31(RPL31), and ribosomal protein S9 (RPL9) are components of the large (60S) human ribosomal subunit. Overexpression of ribosome genes has been shown [37] to promote the translation and protein biosynthesis of Cardiac Allograft Rejection (AR) and cancer-related cytokines. Furthermore, ribosomes in stroke-induced peripheral immunosuppression may be a potential mechanism of sex disparities in outcome following IS [38]. Researchers implicate autoantigens, including RPL7, RPL31, RPL14, and RPL9, in the regulation of diseases such as systemic lupus erythematosus, rheumatoid arthritis, and systemic sclerosis [37]. Herein, we found higher levels of RPL31and RPL9 in IS samples compared to non-IS samples (Figures 8(e) and 8(g)). The IS model had significantly lower RPL7 genes, but there was no significant difference in RPL14 between the two groups (Figures 8(f) and 8(d)).

Our findings suggest a close association of PABPC1, PFDN5, and TNF with female ischemic stroke patients. PABPC1 (poly (A) binding protein cytoplasmic 1) is an important component of the RNA stabilization protein complex involved in germ cell development and mRNA translational regulation, and they have been linked to cancer [39]. Prefoldin (PFDN) is a co-chaperone protein widely accepted to play important roles in normal neuronal development and maintenance [40]. Mounting evidence had demonstrated that [41]inflammation is the principal cause of the pathology and physiology process of IS. Furthermore, the balance between proinflammatory cytokines and anti-inflammatory cytokines was linked to the progression and prognosis of IS patients [42]. Tumor necrosis factor (TNF) is a neuroinflammation cytokine and a potential target in future stroke therapy among the 10 core genes listed above. TNF regulates the size of ischemic injury. Following an ischemic stroke, TNF levels rise in cerebrospinal fluid (CSF) and blood [43]. This is consistent with our research. Additionally, furthermore, there is preliminary evidence that [44] targeting TNF may become a therapeutic approach in ischemic stroke. In addition, previous research has shown [45] a close association of ribosomes with immune cells. The 10 core genes identified in our present research were mostly ribosomal proteins. Therefore, we hypothesize that these genes are linked to T cells, macrophages, NK cells, and neutrophils in female ischemic stroke patients.

In conclusion, this study, for the first time, used microarray samples from IS females for WGCNA. We validated four immune-related gene expression modules and 10 central genes. The findings may be useful in further elucidating the pathogenesis of IS in female patients. Furthermore, we hypothesize that neutrophils and monocytes may play an important role in the disease progression of female IS patients. These findings could point to important biological targets for drug screening and drug design in IS females.

## Figures and Tables

**Table 1:** The primers used in qRT-PCR.

Primers	Forward	Reverse
*β*-actin	5′-TGTCACCAACTGGGACGATA-3′	5′-GGGGTGTTGAAGGTCTCAAA-3′
PRS28	5′-GCTGGCTAGGGTAACTAAAGTGCTG-3′	5′-TCGGATGATAGAGCGGCTGGTG-3′
RPS6	5′-AGCGGTGGGAATGACAAACAAGG-3′	5′-CGCTTCCTCTCTCCAGTTCTCCTAG-3′
RPS15A	5′-ACAGGAAGGTTGAACAAGTGTGGAG-3′	5′-ACAATGAAACCAAACTGCCGTGATG-3′
RPL7	5′-TTGCCCTGAAGACACTGCGAAAG-3′	5′-GCCATCCTAGCCATGCGAATCTC-3′
RPL9	5′-ACACTGGGCTTCCGTTACAAGATG-3′	5′-CAACACCTGTCCTCATCCGAACC-3′
RPL31	5′-TCCTCGGGCACTCAAAGAAATTCG-3′	5′-CTCGGATGCGGTACGGAACATTC-3′
RPL14	5′-TGGAAAGCTGGTCGCAATCGTAG-3′	5′-CGCACTGTGTGGGAACTTGAGG-3′
PABPC1	5′-TACCAGCCAGCACCTCCTTCAG-3′	5′-CAGCGAGGACTTGGTCTTAGTTGAG-3′
PFDN5	5′-GCTGAGGATGCCAAGGACTTCTTC-3′	5′-CATCATTTCCACGACGGCTTGTTTC-3′
TNF	5′-ATGGGCTCCCTCTCATCAGTTCC-3′	5′-ATGGGCTCCCTCTCATCAGTTCC-3′

**Table 2:** The top 10 GO enrichment terms of genes in turquoise module.

ONTOLOGY	ID	Description	p.adjust	Count
BP	GO:0042110	T cell activation	0.000472374	17
BP	GO:1903131	Mononuclear cell differentiation	0.000472374	16
BP	GO:0001819	Positive regulation of cytokine production	0.00120906	15
BP	GO:0002446	Neutrophil mediated immunity	0.003207892	15
BP	GO:0042119	Neutrophil activation	0.003207892	15
BP	GO:1903037	Regulation of leukocyte cell-cell adhesion	0.000472374	14
BP	GO:0030098	Lymphocyte differentiation	0.001054285	14
BP	GO:0007159	Leukocyte cell-cell adhesion	0.001059699	14
BP	GO:0050727	Regulation of inflammatory response	0.001059699	14
BP	GO:0022407	Regulation of cell-cell adhesion	0.003207892	14
CC	GO:0005840	Ribosome	0.003853809	10
CC	GO:0034774	Secretory granule lumen	0.022036358	10
CC	GO:0060205	Cytoplasmic vesicle lumen	0.022036358	10
CC	GO:0031983	Vesicle lumen	0.022036358	10
CC	GO:0022626	Cytosolic ribosome	8.41E-05	9
CC	GO:0044391	Ribosomal subunit	0.003853809	9
CC	GO:0031252	Cell leading edge	0.118712942	9
CC	GO:0005925	Focal adhesion	0.121501769	9
CC	GO:0030055	Cell-substrate junction	0.128157705	9
CC	GO:0045121	Membrane raft	0.006295321	8
MF	GO:0004857	Enzyme inhibitor activity	0.212404345	10
MF	GO:0042277	Peptide binding	0.212404345	9
MF	GO:0033218	Amide binding	0.247781289	9
MF	GO:0003712	Transcription coregulator activity	0.385576533	9
MF	GO:0003735	Structural constituent of ribosome	0.212404345	7
MF	GO:0019207	Kinase regulator activity	0.212404345	7
MF	GO:0019887	Protein kinase regulator activity	0.247781289	6
MF	GO:0030246	Carbohydrate binding	0.389028377	6
MF	GO:0045182	Translation regulator activity	0.247781289	5
MF	GO:0003714	Transcription corepressor activity	0.364059812	5

**Table 3:** The KEGG pathway enrichment analysis of genes in turquoise module.

ID	Description	p.adjust	Count
hsa05171	Coronavirus disease - COVID-19	0.049485195	9
hsa05135	Yersinia infection	0.049485195	7
hsa03010	Ribosome	0.050642504	7
hsa04066	HIF-1 signaling pathway	0.049485195	6
hsa04650	Natural killer cell mediated cytotoxicity	0.08036003	6
hsa04621	NOD-like receptor signaling pathway	0.247330937	6
hsa05202	Transcriptional misregulation in cancer	0.247330937	6
hsa05169	Epstein-Barr virus infection	0.293398065	6
hsa05208	Chemical carcinogenesis - reactive oxygen species	0.327462276	6

## Data Availability

The data used to support the findings of this study are included within the article.

## Supplementary Materials

Supplementary Materials The detailed R code is summarized in the supplementary materials.

## References

[1] Campbell B. C. V., de Silva D. A., Macleod M. R. (2019). Ischaemic stroke. *Nature Reviews. Disease Primers*.

[2] GBD 2016 Neurology Collaborators (2019). Global, regional, and national burden of neurological disorders, 1990-2016: a systematic analysis for the Global Burden of Disease Study 2016. *The Lancet Neurology*.

[3] Ziganshina L. E., Abakumova T., Hoyle C. H. (2020). Cerebrolysin for acute ischaemic stroke. *The Cochrane Database of Systematic Reviews*.

[4] Benjamin E. J., Virani S. S., Callaway C. W. (2018). Correction to: Heart Disease and Stroke Statistics-2018 Update: A Report from the American Heart Association. *Circulation*.

[5] Xu H., Ge Y., Liu Y. (2022). Identification of the key genes and immune infiltrating cells determined by sex differences in ischaemic stroke through co-expression network module. *IET Systems Biology*.

[6] Zou C., Wei C., Wang Z., Jin Y. (2017). Sex differences in outcomes and risk factors among elderly patients with ischemic stroke. *Oncotarget*.

[7] Caprio F. Z., Sorond F. A. (2019). Cerebrovascular disease: primary and secondary stroke prevention. *The Medical Clinics of North America*.

[8] Qiu X., Lin J., Liang B., Chen Y., Liu G., Zheng J. (2021). Identification of hub genes and microRNAs associated with idiopathic pulmonary arterial hypertension by integrated bioinformatics analyses. *Frontiers in Genetics*.

[9] Goldstein L. B., Adams R., Becker K. (2001). Primary prevention of ischemic stroke. *Stroke*.

[10] Li Z., Cui Y., Feng J., Guo Y. (2020). Identifying the pattern of immune related cells and genes in the peripheral blood of ischemic stroke. *Journal of Translational Medicine*.

[11] Chen G., Li L., Tao H. (2021). Bioinformatics identification of ferroptosis-related biomarkers and therapeutic compounds in ischemic stroke. *Frontiers in Neurology*.

[12] Feng B., Meng X., Zhou H. (2021). Identification of dysregulated mechanisms and potential biomarkers in ischemic stroke onset. *International Journal of General Medicine*.

[13] Chamankhah M., Eftekharpour E., Karimi-Abdolrezaee S., Boutros P. C., San-Marina S., Fehlings M. G. (2013). Genome-wide gene expression profiling of stress response in a spinal cord clip compression injury model. *BMC Genomics*.

[14] Langfelder P., Horvath S. (2008). WGCNA: an R package for weighted correlation network analysis. *BMC Bioinformatics*.

[15] Szklarczyk D., Gable A. L., Lyon D. (2019). STRING v11: protein-protein association networks with increased coverage, supporting functional discovery in genome-wide experimental datasets. *Nucleic Acids Research*.

[16] Wang Y., Wang X., Zhang X. (2020). D1 receptor-mediated endogenous tPA upregulation contributes to blood-brain barrier injury after acute ischaemic stroke. *Journal of Cellular and Molecular Medicine*.

[17] Miller E. C., Leffert L. (2020). Stroke in pregnancy. *Anesthesia and Analgesia*.

[18] Elgendy I. Y., Gad M. M., Mahmoud A. N., Keeley E. C., Pepine C. J. (2020). Acute stroke during pregnancy and puerperium. *Journal of the American College of Cardiology*.

[19] Carvalho T. F. M., Silva J. C. F., Calil I. P., Fontes E. P. B., Cerqueira F. R. (2017). Rama: a machine learning approach for ribosomal protein prediction in plants. *Scientific Reports*.

[20] Agarwal A., Park S., Ha S. (2020). Quantitative mass spectrometric analysis of the mouse cerebral cortex after ischemic stroke. *PLoS One*.

[21] Yilmaz G., Arumugam T. V., Stokes K. Y., Granger D. N. (2006). Role of T lymphocytes and interferon-*γ* in ischemic stroke. *Circulation*.

[22] Del Zoppo G. J., Schmid-Schönbein G. W., Mori E., Copeland B. R., Chang C. M. (1991). Polymorphonuclear leukocytes occlude capillaries following middle cerebral artery occlusion and reperfusion in baboons. *Stroke*.

[23] Rosell A., Cuadrado E., Ortega-Aznar A., Hernández-Guillamon M., Lo E. H., Montaner J. (2008). MMP-9-positive neutrophil infiltration is associated to blood-brain barrier breakdown and basal lamina type IV collagen degradation during hemorrhagic transformation after human ischemic stroke. *Stroke*.

[24] Herz J., Sabellek P., Lane T. E., Gunzer M., Hermann D. M., Doeppner T. R. (2015). Role of neutrophils in exacerbation of brain injury after focal cerebral ischemia in hyperlipidemic mice. *Stroke*.

[25] Soderquest K., Walzer T., Zafirova B. (2011). Cutting edge: CD8+ T cell priming in the absence of NK cells leads to enhanced memory responses. *Journal of Immunology (Baltimore, Md. : 1950)*.

[26] Gan Y., Liu Q., Wu W. (2014). Ischemic neurons recruit natural killer cells that accelerate brain infarction. *Proceedings of the National Academy of Sciences of the United States of America*.

[27] Wattananit S., Tornero D., Graubardt N. (2016). Monocyte-derived macrophages contribute to spontaneous long-term functional recovery after stroke in mice. *The Journal of neuroscience : the official journal of the Society for Neuroscience*.

[28] Cihakova D., Barin J. G., Afanasyeva M. (2008). Interleukin-13 protects against experimental autoimmune myocarditis by regulating macrophage differentiation. *The American Journal of Pathology*.

[29] McGeer P. L., McGeer E. G. (2011). History of innate immunity in neurodegenerative disorders. *Frontiers in Pharmacology*.

[30] Wang Y., Zhang J. H., Sheng J., Shao A. (2019). Immunoreactive cells after cerebral ischemia. *Frontiers in Immunology*.

[31] Xie X., Guo P., Yu H., Wang Y., Chen G. (2018). Ribosomal proteins: insight into molecular roles and functions in hepatocellular carcinoma. *Oncogene*.

[32] Wang W., Nag S., Zhang X. (2015). Ribosomal proteins and human diseases: pathogenesis, molecular mechanisms, and therapeutic implications. *Medicinal Research Reviews*.

[33] Li M. Y., Fan L. N., Han D. H. (2020). Ribosomal S6 protein kinase 4 promotes radioresistance in esophageal squamous cell carcinoma. *The Journal of Clinical Investigation*.

[34] Kim S., Jang Y. H., Chau G. C., Pyo S., Um S. H. (2013). Prognostic significance and function of phosphorylated ribosomal protein S6 in esophageal squamous cell carcinoma. *Modern Pathology*.

[35] Xiao H., Wang H., Silva E. A. (2015). The Pallbearer E3 ligase promotes actin remodeling via RAC in efferocytosis by degrading the ribosomal protein S6. *Developmental Cell*.

[36] Wei J., Kishton R. J., Angel M. (2019). Ribosomal proteins regulate MHC class I peptide generation for immunosurveillance. *Molecular Cell*.

[37] Woolford J. L., Baserga S. J. (2013). Ribosome biogenesis in the yeast Saccharomyces cerevisiae. *Genetics*.

[38] Xie J. Q., Lu Y. P., Sun H. L. (2020). Sex difference of ribosome in stroke-induced peripheral immunosuppression by integrated bioinformatics analysis. *BioMed Research International*.

[39] Su R., Ma J., Zheng J. (2020). PABPC1-induced stabilization of BDNF-AS inhibits malignant progression of glioblastoma cells through STAU1-mediated decay. *Cell Death & Disease*.

[40] Lee Y., Smith R. S., Jordan W. (2011). Prefoldin 5 Is Required for Normal Sensory and Neuronal Development in a Murine Model. *The Journal of Biological Chemistry*.

[41] Duris K., Splichal Z., Jurajda M. (2018). The role of inflammatory response in stroke associated programmed cell death. *Current Neuropharmacology*.

[42] Dénes A., Ferenczi S., Kovács K. J. (2011). Systemic inflammatory challenges compromise survival after experimental stroke via augmenting brain inflammation, blood- brain barrier damage and brain oedema independently of infarct size. *Journal of Neuroinflammation*.

[43] Lambertsen K. L., Finsen B., Clausen B. H. (2019). Post-stroke inflammation-target or tool for therapy?. *Acta Neuropathologica*.

[44] Clausen B. H., Wirenfeldt M., Høgedal S. S. (2020). Characterization of the TNF and IL-1 systems in human brain and blood after ischemic stroke. *Acta Neuropathologica Communications*.

[45] Dhanesha N., Patel R. B., Doddapattar P. (2022). PKM2 promotes neutrophil activation and cerebral thromboinflammation: therapeutic implications for ischemic stroke. *Blood*.

